# Supplementary material for: Low-dimensional encoding of decisions in parietal cortex reflects long-term training history
Source: Nat Commun. 2023 Feb 23;14:1010. doi: 10.1038/s41467-023-36554-5 (PMC9950136; doi:10.1038/s41467-023-36554-5)
Supplement: Supplementary file 1 — Supplementary Information [file 41467_2023_36554_MOESM1_ESM.pdf]

Supplementary Material: Low-dimensional encoding of decisions in parietal cortex reflects long-term training history

Authors: Kenneth W. Latimer[1*] & David J. Freedman[1]
[1]Department of Neurobiology, University of Chicago
[*]Correspondence; E-mail: latimerk@uchicago.edu.

January 23, 2023

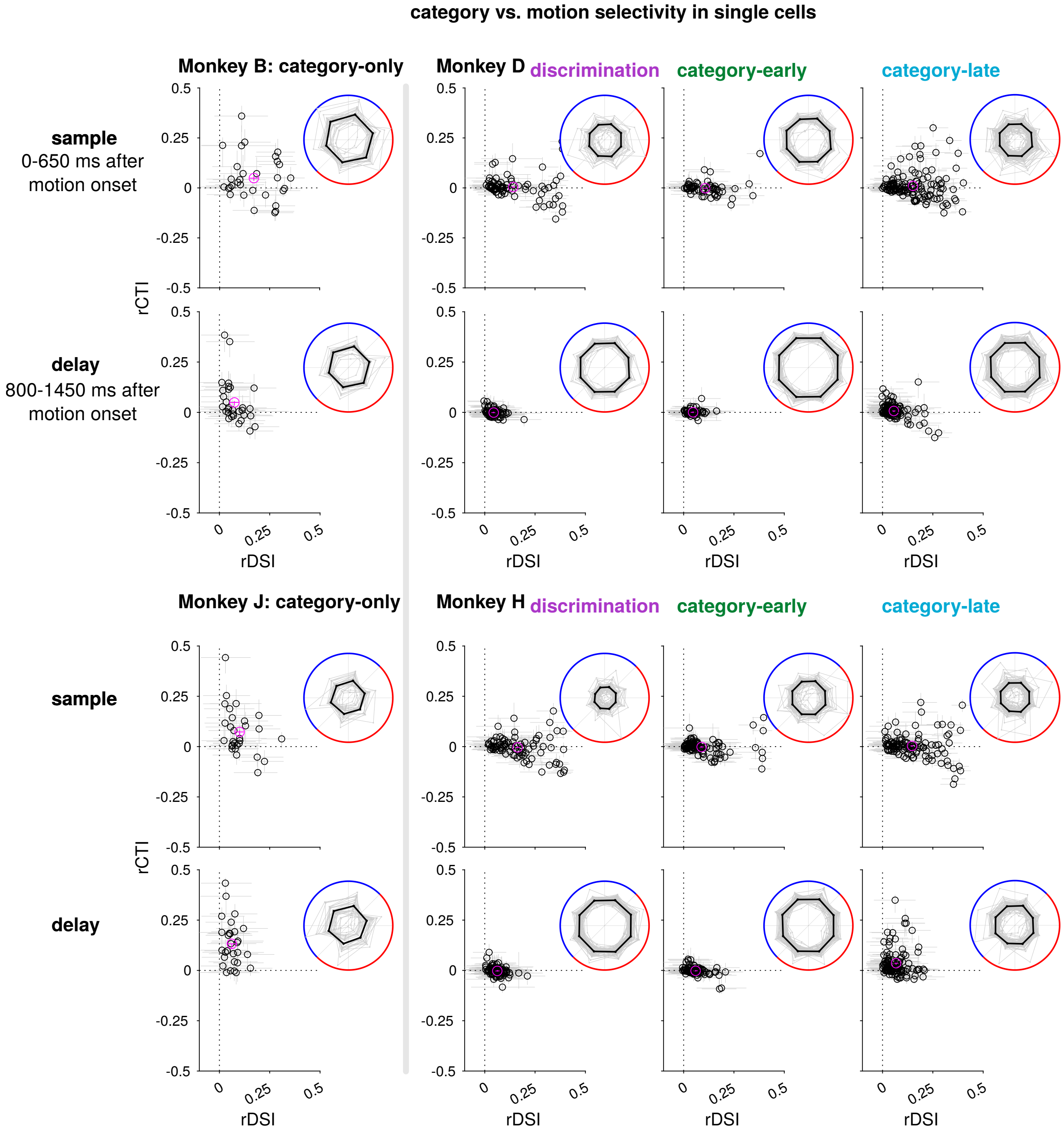

Supplementary Figure 1: Category and direction selectivity measured by rCTI and rDSI for each individual cell in all eight populations during the sample (top rows) and delay periods (bottom rows). The points denote the mean over bootstraps and the error bars show a 95% interval. The magenta points show the population means. The polar plot inserts show the tuning curves (normalized to mean unit firing rate), and the black trace shows the population average. Category and direction are correlated. As a result, some single cells may be identified as having category tuning during the discrimination task.

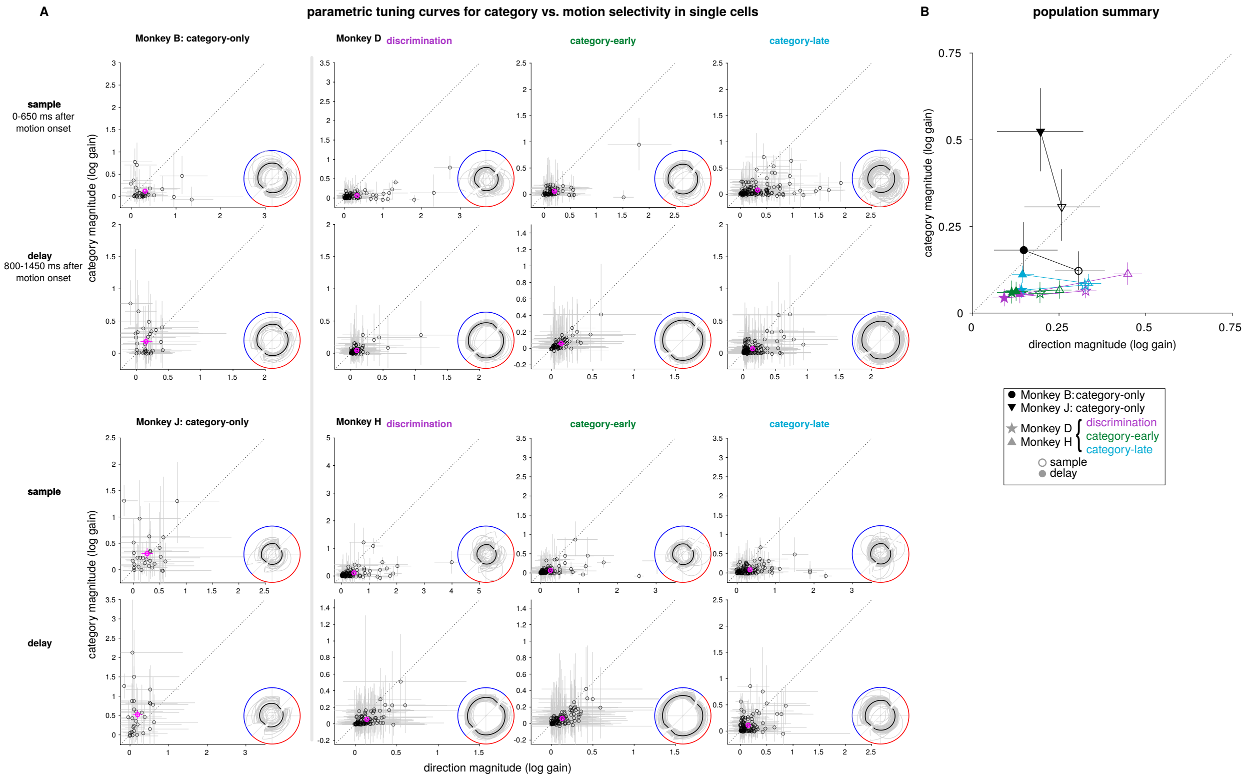

Supplementary Figure 2: Category and direction selectivity measured by parametric tuning curves. **a**, The category and direction selectivity estimated for each individual cell in all eight populations during the sample (top rows) and delay periods (bottom rows). The points denote the mean over bootstraps and the error bars show a 95 % interval. The magenta points show the population means. The polar plot inserts show the fit tuning curves (normalized to mean unit firing rate), and the black trace shows the population average. Because the positive restriction on tuning magnitudes can produce a positive bias in the estimated tuning, the magnitudes were bias-corrected by subtracting the mean direction or category magnitude fit to shuffled data (as a result, the error bars sometimes go below zero to indicate that the tuning was not significantly larger than expected by chance; see Methods). **b**, The population mean category- and direction-selectivity for the sample (open symbols) and delay (filled symbols) periods. The error bars show a 95 % region of the population mean across bootstraps.

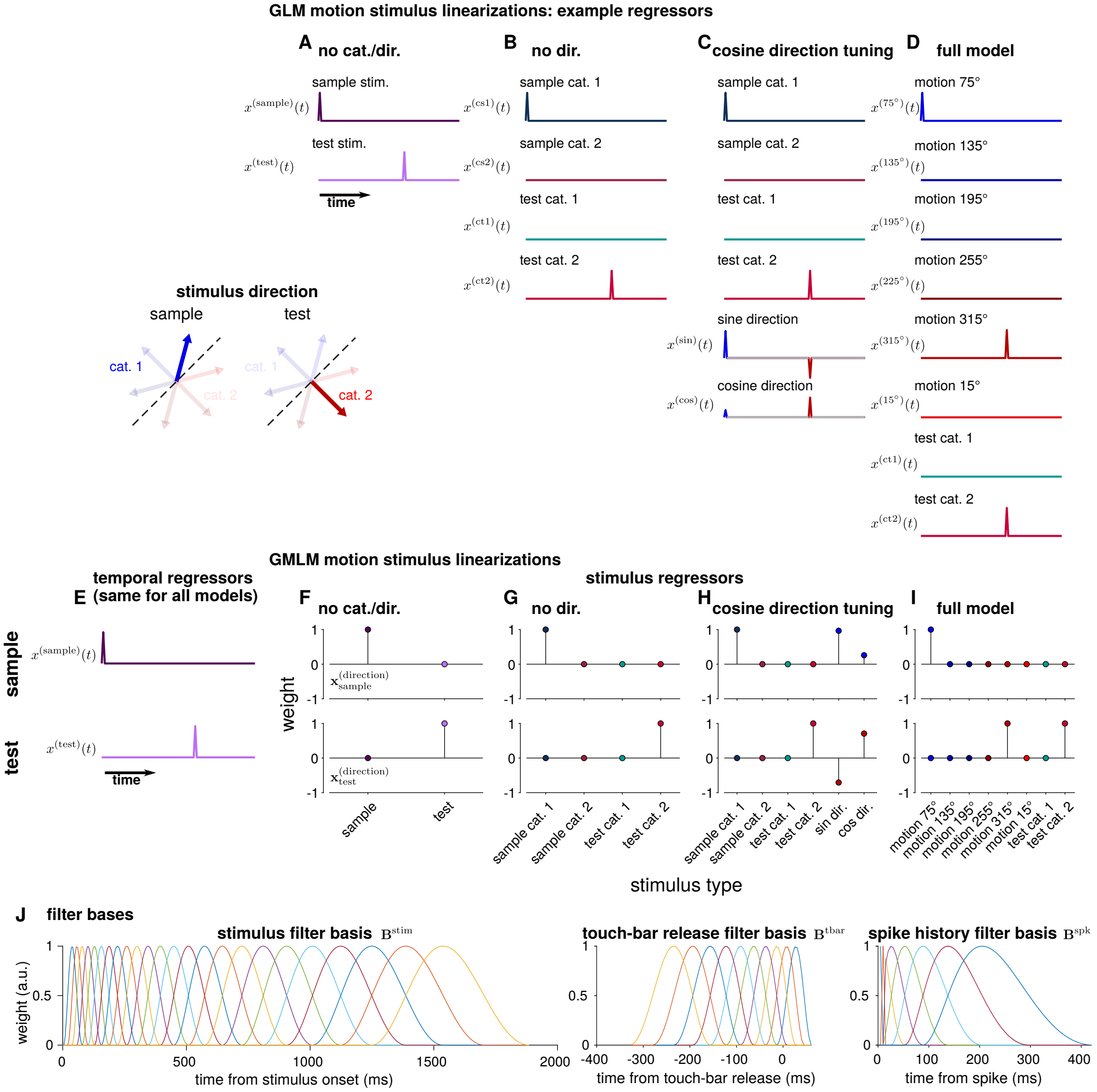

Supplementary Figure 3: Linearizations of the discrimination or categorization tasks in the GMLM. **a-d**, The temporal event regressors for the four GLM types for an example trial with a sample stimulus 75° (category one) and test stimulus of 315° (category two). **a**, The two stimulus events for the no category or direction tuning model. The top event is 1 at the sample stimulus onset time and 0 elsewhere, and the bottom event is 1 at the test stimulus onset time and 0 elsewhere. **b**, The stimulus events for the no direction tuning model. The two sample (or test) category events encode the onset time of a sample (or test) stimulus only for a specific category (the sample category two event is 0 for this trial because the sample stimulus is category one). **c**, The stimulus events for the cosine tuning model. The category events are the same as the category events in **b**. The sine (or cosine) event is equal to the sine (or cosine) of the stimulus direction at the onset of either stimulus. **d**, The stimulus events for the full tuning model. The first six events are 1 at the onset time of a specific stimulus direction (sample or test). The two category events are the same as before. This configuration is identifiable while allowing the category tuning to be different between the sample and test period, while keeping the direction tuning constant. **e-i**, The GMLM regressors for the set of nested models. **e**, The GMLM temporal events for the sample (top) and test (bottom) stimulus onset times. **f-i**, The GMLM stimulus weightings for the four model configurations for the sample (top) and test (bottom) stimulus correspond to the weight of the stimulus events in **a-d**. The complete temporal kernels in the corresponding GLMs are thus the outer product of the temporal regressors in E and the weights of the weights in F-I, summed over the sample and test events. **j**, The three bases for the temporal kernels used in both the GLM and GMLM: the stimulus event bases (left), the touch-bar release basis (middle), and the spike history basis (right). The bases were orthonormalized for model fitting.

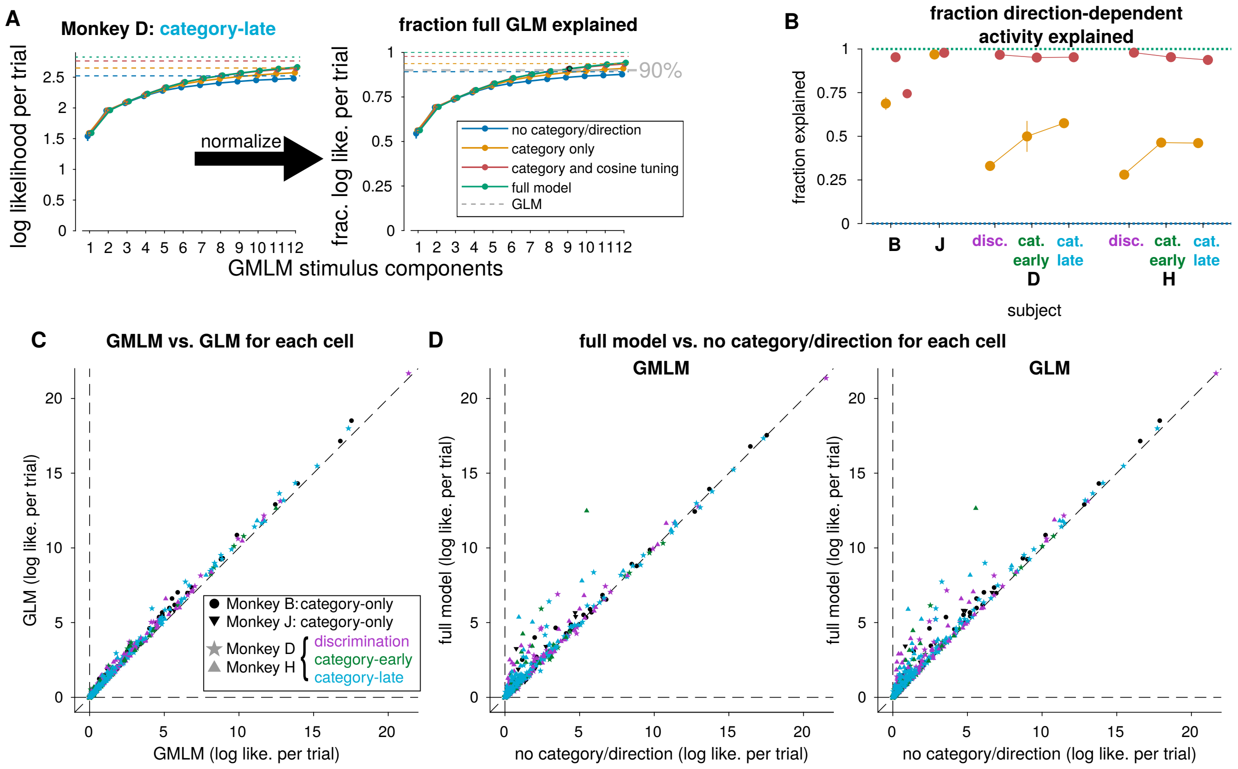

Supplementary Figure 4: Selecting the number of components to include in the GMLM confirms that the population activity is low dimensional (i.e., that it can be well-summarized with a small number of components). **a**, The mean cross-validated log likelihood per trial averaged across neurons of the GMLM as a function of the stimulus kernel tensor rank was computed relative to the model without any stimulus terms (i.e., the rank 0 model) for each LIP populations. (left) Each trace shows the log likelihood for a single model parameterization fit to the monkey D, category-late population: no stimulus information (i.e., only mean temporal dynamics; blue), category only (no specific direction information; yellow), category plus cosine direction tuning (red), and the full model with flexible direction tuning (green). The dashed lines show the mean cross-validated likelihood of the GLMs, which correspond to the full-rank model. (right) The fraction of log likelihood of the full GLM (fraction of the log likelihood that could be captured by the GMLM) was used to select the GMLM rank. This fraction is the cross-validated log likelihood divided by the log likelihood of the full GLM (dashed green line), and the threshold for rank selection was 90 % of the log likelihood per trial. Supplementary Fig. 11 shows that direction tuning was the same across sample and test stimuli. This is similar to the pseudo-R^2 measure (a quantity which is comparable to variance explained in linear-Gaussian models but is more appropriate for models like the GMLM which use spike counts; see [80]) but we have used the GLM as the "saturated" model. **b**, The difference between the no category or direction model (blue in part A left) and the full model (green in part A left) differed across populations, we quantified the fraction of direction-dependent activity explained by each model as the normalized log-likelihood explained between the no category or direction model and the full model (at the selected ranks of the GMLM). After this scaling, the no direction or category model (blue) explains 0 % of the direction-dependent activity, while the full model explained 100 % of the direction-dependent activity. The category-only model (orange) is then compared against the category and cosine-tuning model (red). **c**, The fit of the GMLMs compared to the GLMs for each individual cell. The axes show the model's cross-validated log likelihood per trial (over the null model without any stimulus tuning). **d**, The improvement of the full-tuning model over the model with no direction or category tuning for each cell. The GMLM fits (left) shows similar improvement to the GLMs (right).

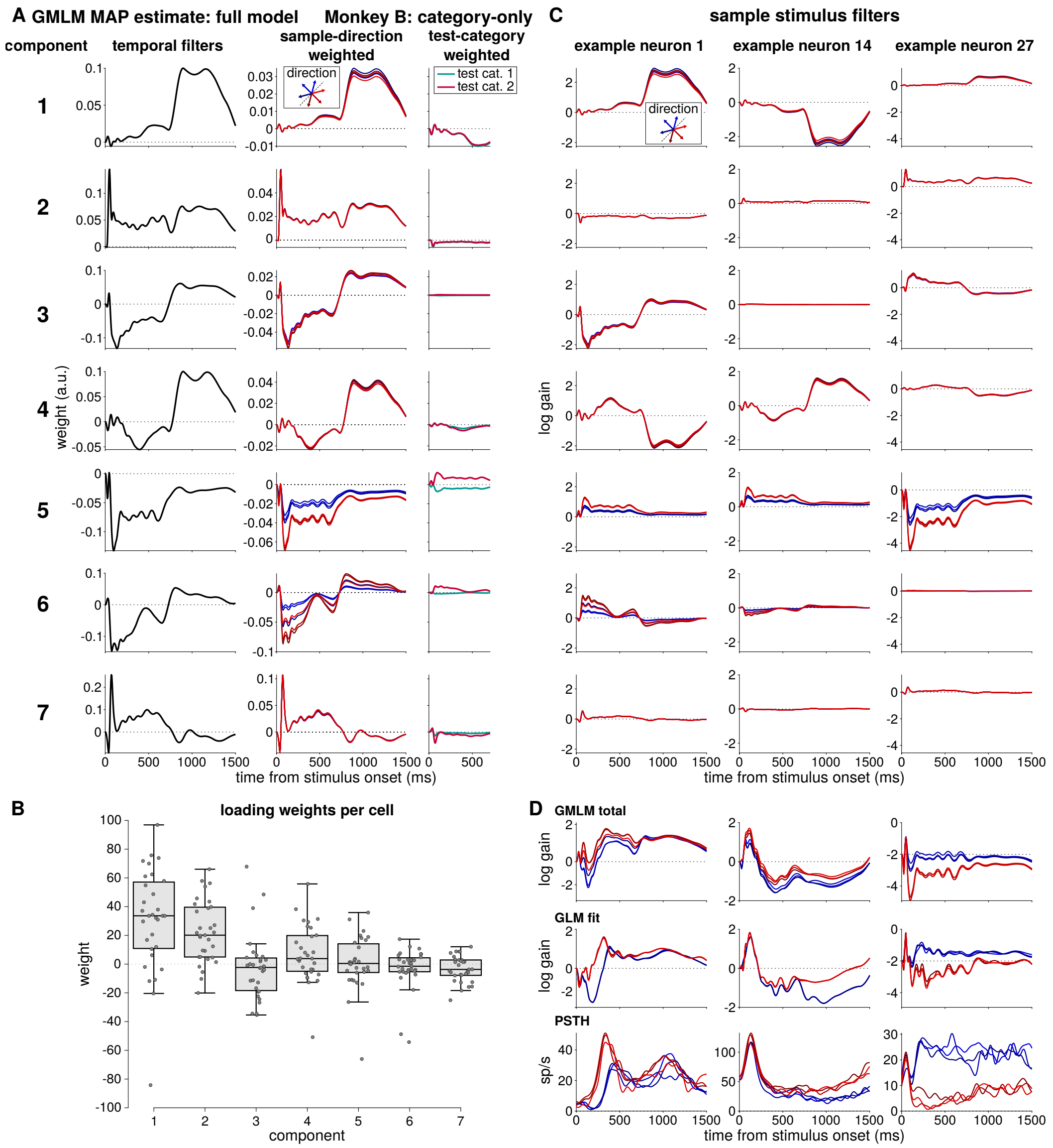

Supplementary Figure 5: GMLM fit to a population of LIP neurons recorded during the categorization task. **a**, The seven stimulus components of the GMLM fit to data from monkey B are given in each row. (left) The temporal (normalized) kernels. (middle) The temporal kernels weighted for each sample stimulus direction. (right) The temporal kernels weighted by the test category. The total kernels for each test direction are computed by adding the test category kernels to the sample direction filter. Different components can have different temporal response dynamics and different stimulus tuning properties: for example, component 5 shows strong differentiation between the two stimulus categories (red and blue), while component 2 does not. In this model fit, only a couple of components show motion direction or category selectivity (5 and 6). This sparsity of direction encoding is consistent with low-dimensional encoding of the direction or category, where the temporal response to different category and directions are similar. **b**, The distributions of weights for each component across all neurons (dots). The components are shown ordered by the mean squared weight. Like with PCA, the first components have on average a higher weight in each neuron than the last. The box plots show the median and 25 and 75 % quartiles over neurons and the whiskers extend to a 1.5 interquartile range from the edges ($n = 31$). **c**, Each column shows the model fit of the sample stimulus kernels for three example neurons. The first seven rows show the components scaled by each neuron's weighting of the component (the middle column of part A with neuron-dependent scaling from **b**). **d**, Total sample stimulus tuning for the three example cells. The total GMLM stimulus kernels (i.e., the sum of rows in **c**; top row) compared with the single-cell GLM fits (middle), and the sample-direction conditioned PSTHs (bottom). We note that the goal of the GMLM is not to exactly fit each individual cell - but instead it extracts the main stimulus-dependent responses that capture the entire population. The touch-bar related components are shown in Supplementary Fig. 6a

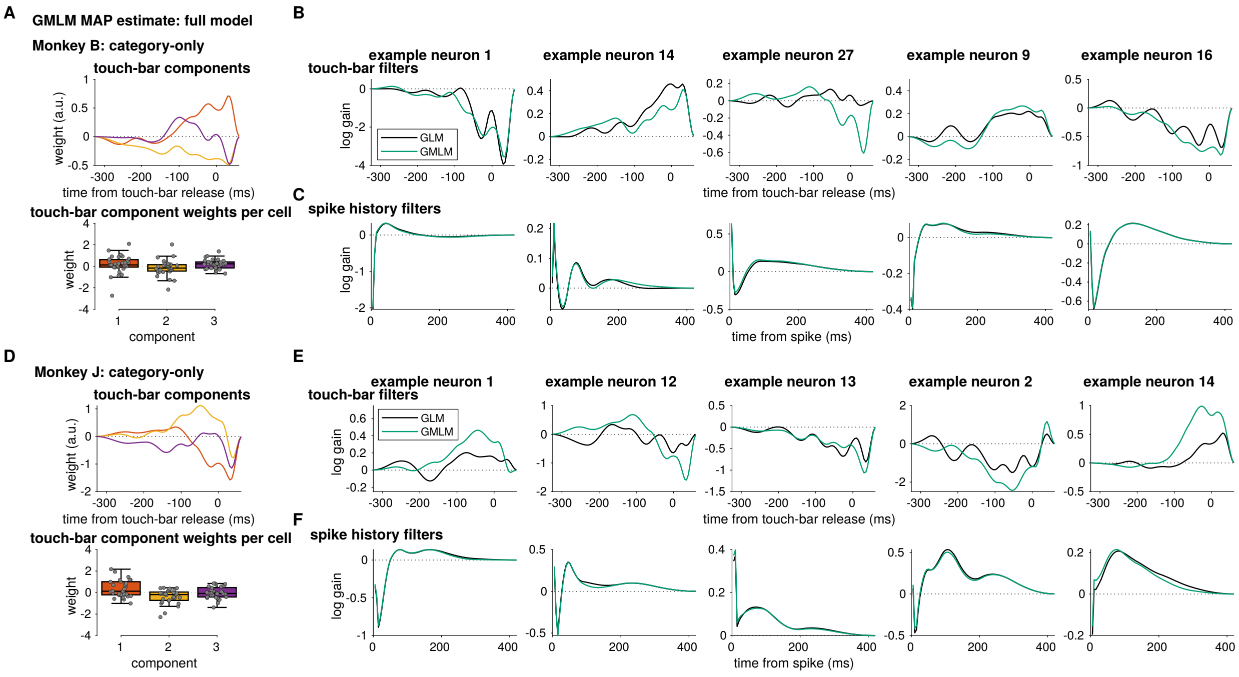

Supplementary Figure 6: Touch-bar and spike-history kernels from the GMLM (full model) and the GLM fits. (**A**) The low-dimensional touch-bar release components for monkey B. (top) The three temporal kernels. (bottom) The loading weights for each touch-bar release component for each cell (points). The box plots show the median and 25 and 75 % quartiles over neurons and the whiskers extend to a 1.5 interquartile range from the edges. **b**, Example touch-bar filters for five cells. The GLM touch-bar filters (black) are compared to the GMLM fit (cyan). **c**, Spike-history filters fit to the same cells in **b**. The GLM spike-history filters (black) are nearly identical to the to the GMLM fit (cyan). **d-f**, Same as **a-c** for monkey J ($n = 29$ cells).

GMLM fits: low-dimensional responses to sample stimulus with mean response

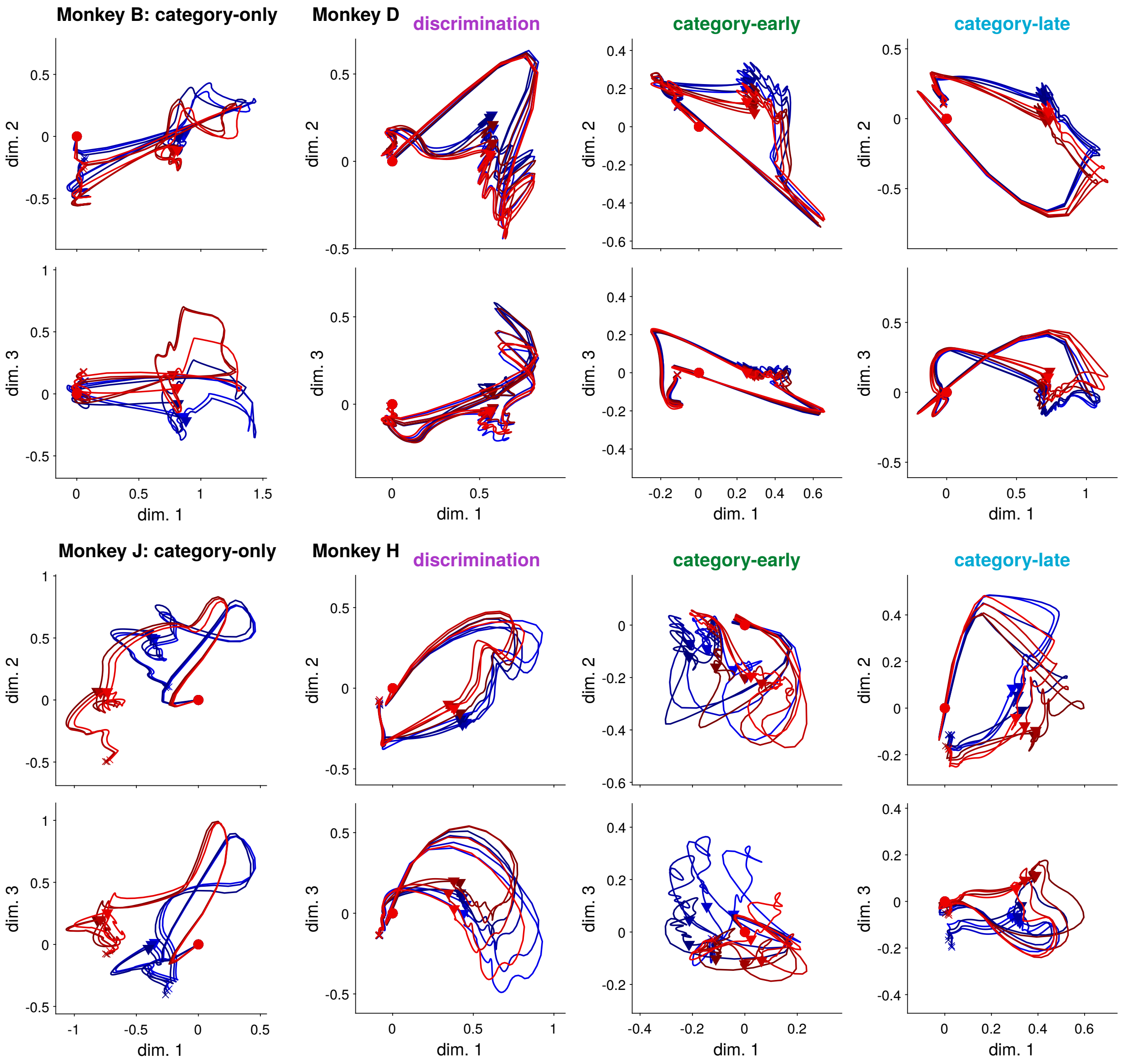

Supplementary Figure 7: The low-dimensional responses reflect strong category and motion independent components. The top three dimensions of the GMLM subspaces (full model) in response to the sample stimulus for each animal and recording epoch without removing the mean over motions directions.

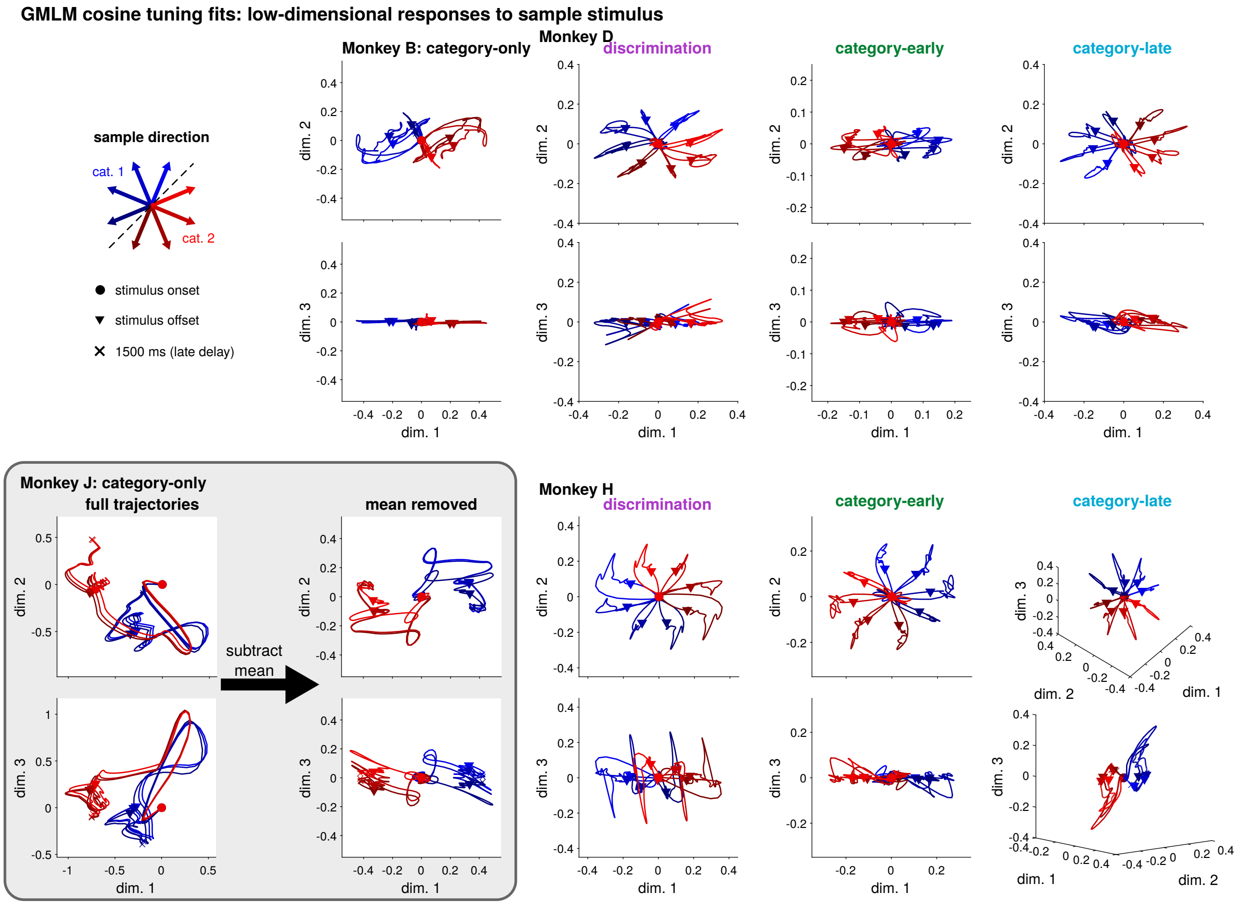

Supplementary Figure 8: Separating cosine-direction tuning and category in the cosine-tuned GMLM. Each of the top three dimensions (with the mean removed) for each LIP population are shown as a function of time relative to sample stimulus onset. For the cosine model, we can separate the direction and category components. The left column for each population shows the sample category trajectories in the three dimensions. The right columns shows the direction trajectories, decoupled from category.

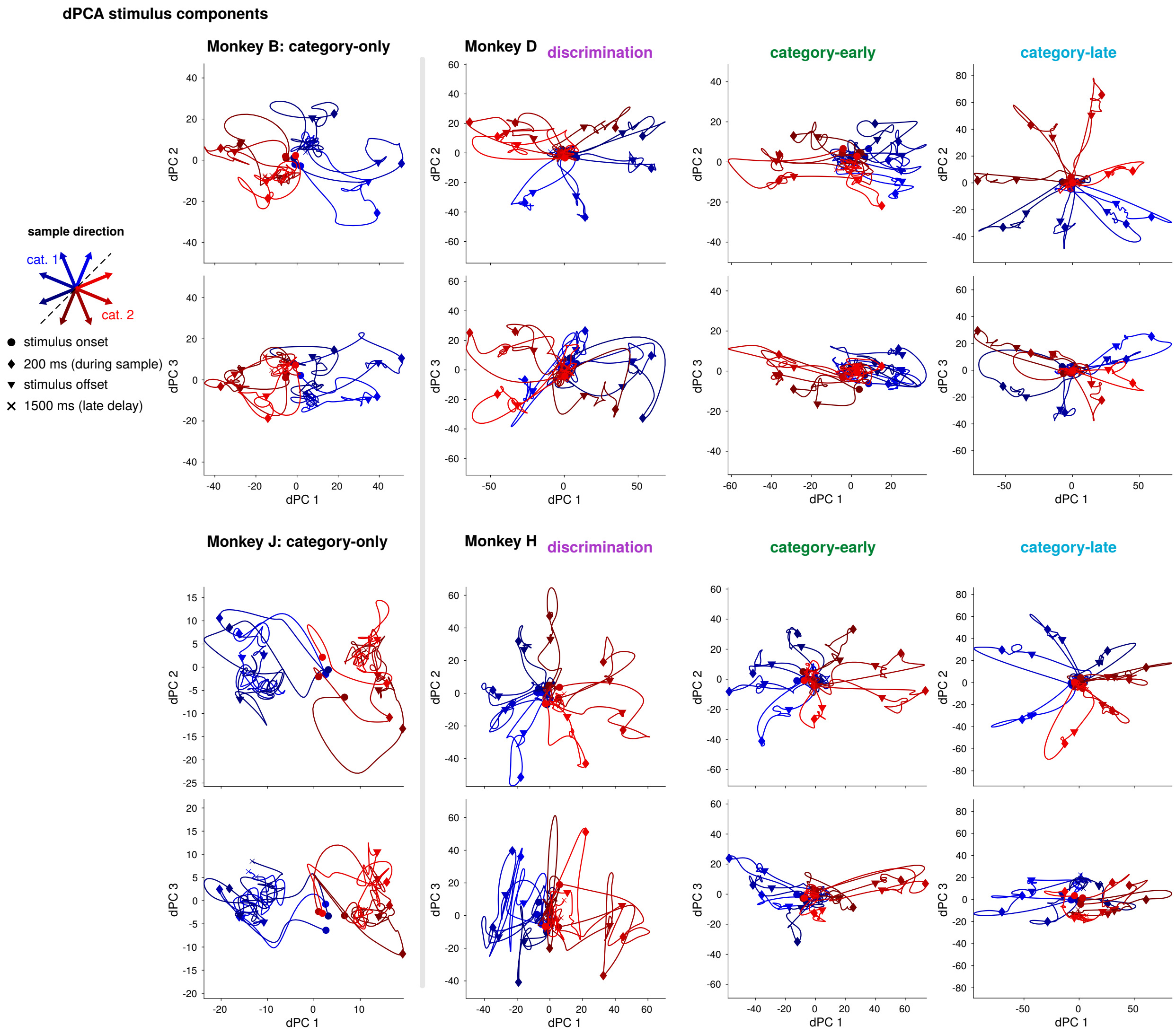

Supplementary Figure 9: demixed PCA space for all eight LIP populations. Each pair of plots shows the top three demixed PCs explaining stimulus direction and category dependent responses. Each trace shows the population response to one sample stimulus direction (denoted by color) from sample onset to 1500 ms after stimulus onset (near the end of the delay period). The markers indicate stimulus onset and offset.

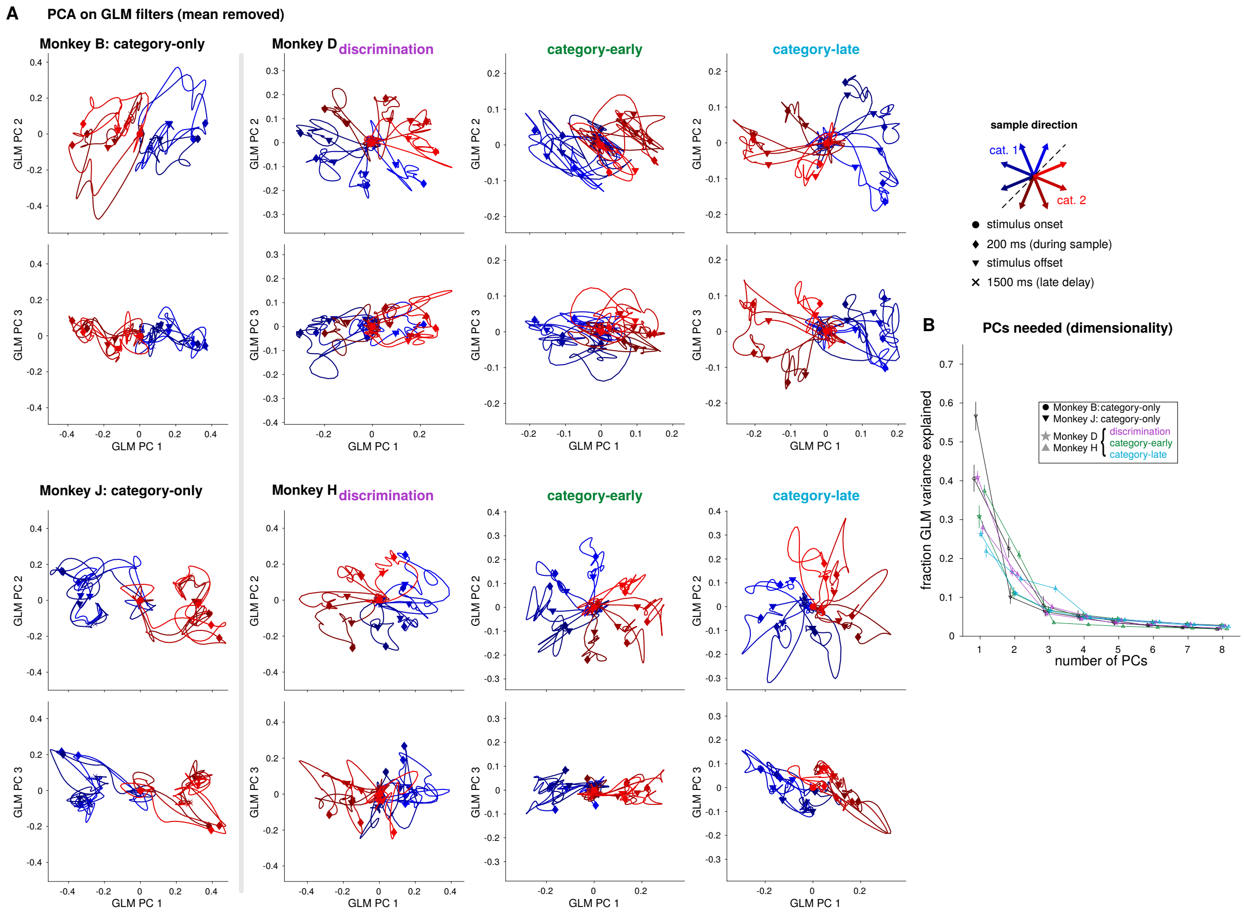

Supplementary Figure 10: We performed PCA on the GLM fits to each neuron using the model's filters for each of the sample stimulus direction. The mean response across all directions for each neuron was subtracted before performing PCA. **a**, Each pair of plots shows the top three PCs explaining stimulus direction and category dependent responses. Each trace shows the population response to one sample stimulus direction (denoted by color) from sample onset to 1500 ms after stimulus onset (near the end of the delay period). The markers indicate stimulus onset and offset. **b**, Fraction of variance of the (mean-removed) filters captured by each PC. The direction-dependent response showed very low dimensions: only two or three PCs captured most of the variance in the model fits. The points denote the median over bootstraps and the error bars show a 95 % region ($n = 1000$ bootstraps)

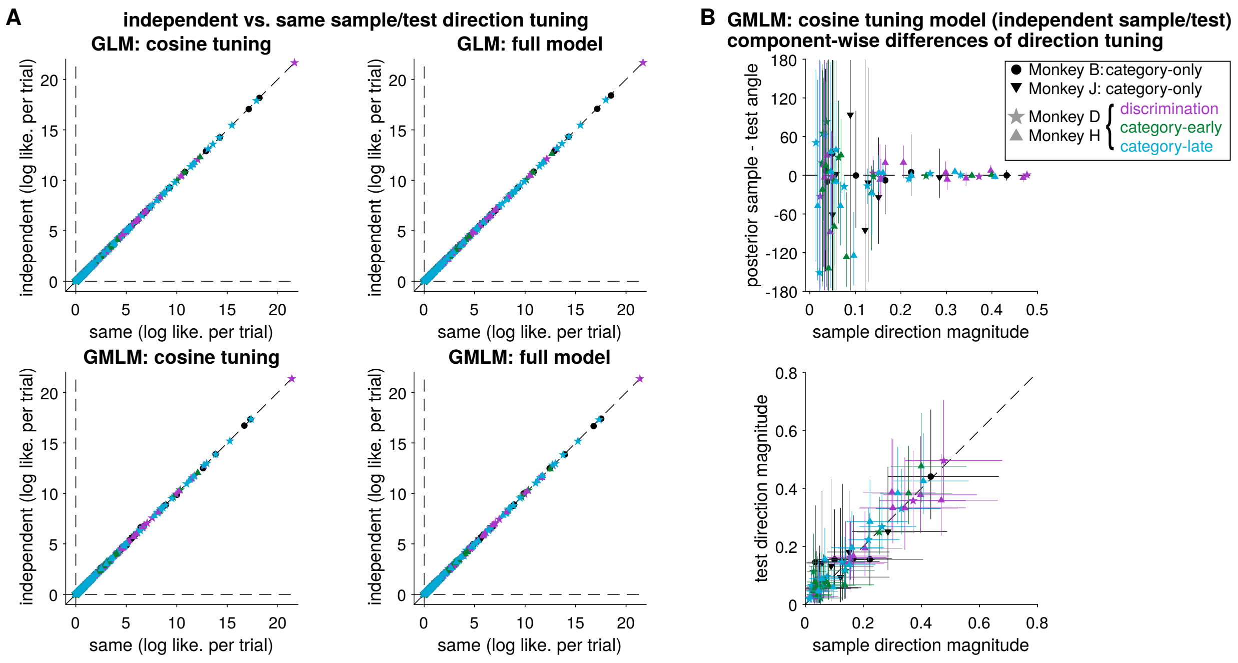

Supplementary Figure 11: The GMLM and GLM find similar direction tuning across the sample and test stimuli. **a**, cross-validated per-trial log likelihoods for each cell (relative to the GMLM without any stimulus terms, $R_s = 0$). The left column shows comparisons using the cosine tuning model and the right column shows the full model. The top row compares the GLM fits to single cells and the bottom row shows the GMLM fits for the same model configurations. No population showed a significant improvement including the independent directions (for each model and population $p > 0.8$, one-sided Wilcoxin signed rank test with Benjamini-Hochberg correction). Several populations indicated that overfitting occurred with the GLM with independent directions: the same direction model was on average better. **b**, Bayesian analysis of the cosine-tuned GMLM with independent sample and test direction parameters. Each point represents a single GMLM stimulus component for one population (i.e., there are seven points for monkey B because we selected seven GMLM stimulus components). (top) The posterior difference in preferred angle between the sample and test stimuli as a function of the magnitude of sample direction tuning. The angle is $\theta^{(r)}$ and the magnitude is $a^{(r)}$ in Eq. 61 (see Methods). As the magnitude increases, the test and sample directions tend towards zero. At lower magnitudes, the preferred angle is difficult to estimate (undetectable) and therefore the difference shows high uncertainty. (bottom) The magnitude of the sample and test direction tuning for each component. Points show the posterior median and error bars show 99 % credible intervals.

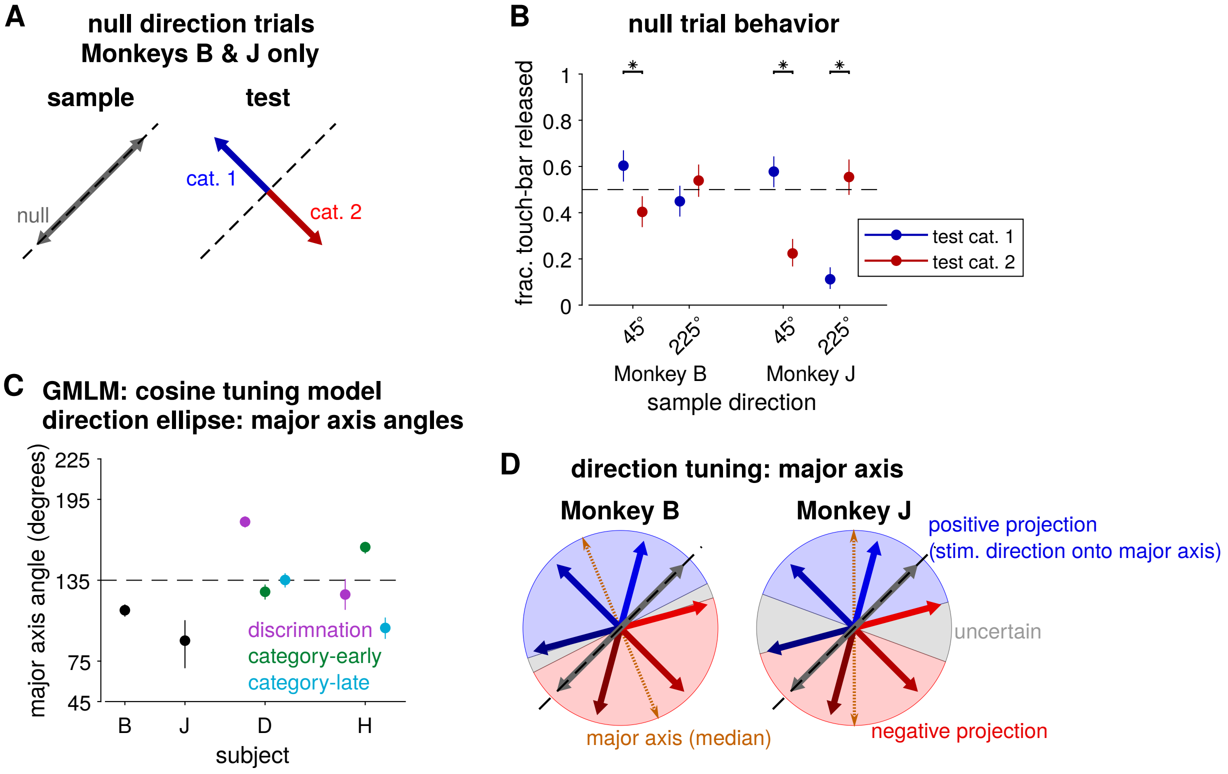

Supplementary Figure 12: Analysis of the direction tuning of the low-dimensional GMLM cosine tuning model components. **a**, The sample and test directions selected on "null" direction trials for monkeys B and J. The sample directions lie on the category boundary. These trials were not included in the GMLM analysis and were rewarded randomly. **b**, Behavior for the four possible combinations of angles on the null-direction trials. The color indicates the test direction/category. The points show the posterior mean estimate of the fraction of touch-bar released during the test stimulus presentation (see Methods: Behavioral performance for details of the Bayesian model) and the error bars denote a 99 % credible interval. Asterisks indicate that the response proportion for the two test directions was different for a given null sample direction ($p < 0.01$; two-sided Wilcoxon rank sum test, Holm-Bonferroni corrected; Monkey B 45° $n = 341, 350$ test cat 1 and test cat 2 trials respectively, $p = 1.25 \times 10^{-7}$; B 225° $n = 363, 332$, $p = 1.77 \times 10^{-2}$; J 45° $n = 358, 320$, $p = 4.94 \times 10^{-21}$; J 225° $n = 293, 274$, $p = 1.13 \times 10^{-29}$). **c**, Bayesian analysis of the cosine tuned GMLM. The GMLM defines the direction tuning as an ellipse in a low-dimensional space. We computed the angle of the major axis of the ellipse: the angle with the most modulation in the low-dimensional space (see Methods Eq. 59). The angle is only identifiable up to 180°. Therefore, we placed it within 45° to 225° to align with the task. If the axis aligned exactly with the categorization task, the angle would be 135°. The ellipse depends on time relative to stimulus onset, and so we took the mean angle during the first 650 ms of stimulus presentation. The points show the posterior median and the error bars denote a 99 % credible interval (50 000 posterior samples). **d**, Illustration of how the direction ellipse's major axis aligns with the task directions. The blue region shows where motion-direction angles project positively along the major axis vector (generally overlapping with category one). The red region shows where motion-direction angles project negatively along the major axis vector. The gray region shows angles that are within the 99 % credible region of the posterior (from C) and cannot be classified. We note that the regions do not exactly align with the category bounds. However, they do correlate with the monkeys' choice biases for the null directions: for monkey B, the 45° null-direction (up and to the right) is in the blue region and the monkey was more likely to release the touch-bar on 45° trials when the test stimulus was in category one than for a category two test stimulus.

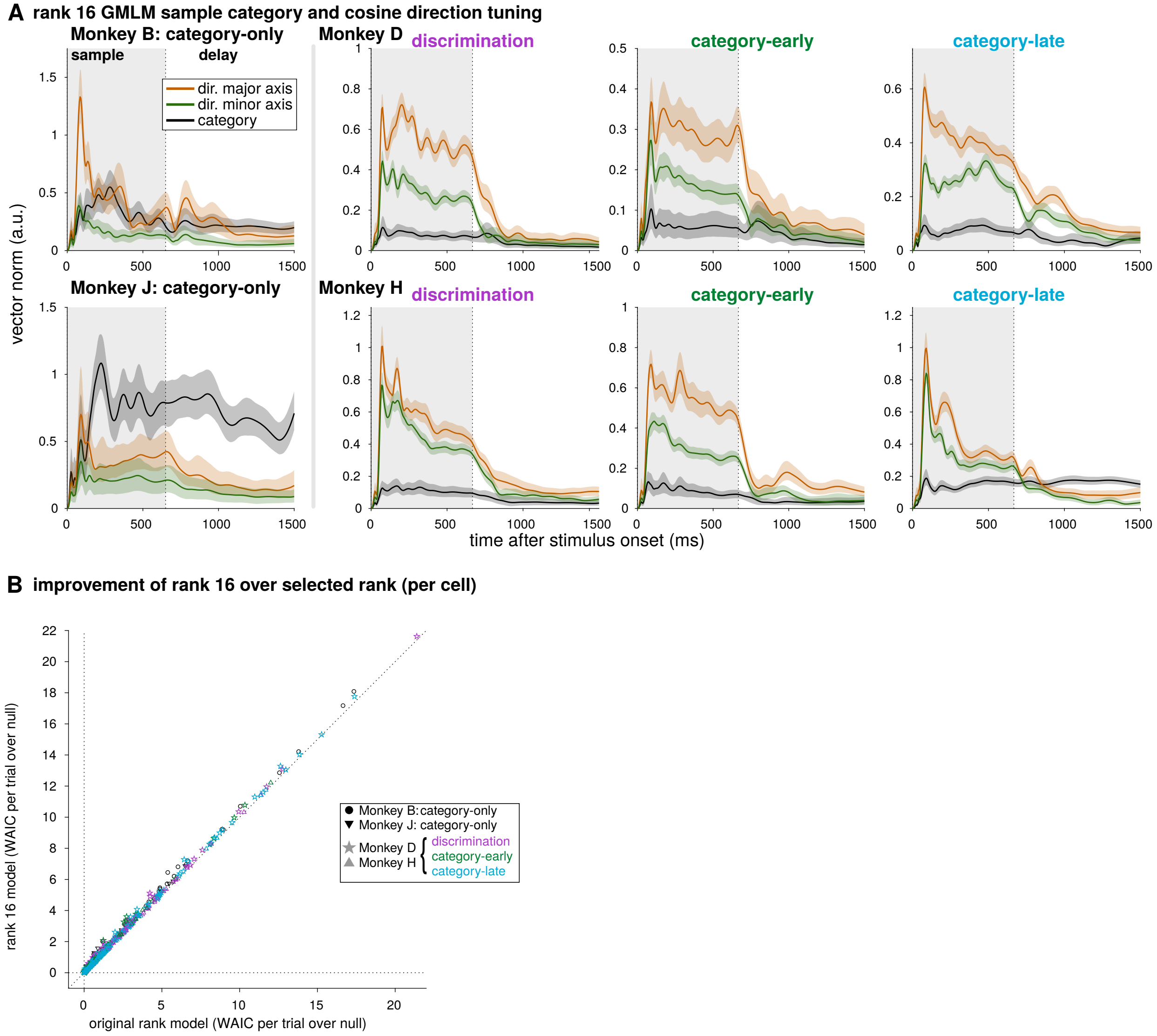

Supplementary Figure 13: Increasing the components in the GMLM to 16 shows similar results to the lower-rank models selected by cross-validation. **a**, The same as Fig. 7b for the rank-16 model model. The solid lines denote the posterior median at each time point, and the shaded regions denote 99 % credible intervals. **b**, Model comparison of the rank 16 GMLM with the selected rank (higher WAIC values indicates the preferred model) for each cell.

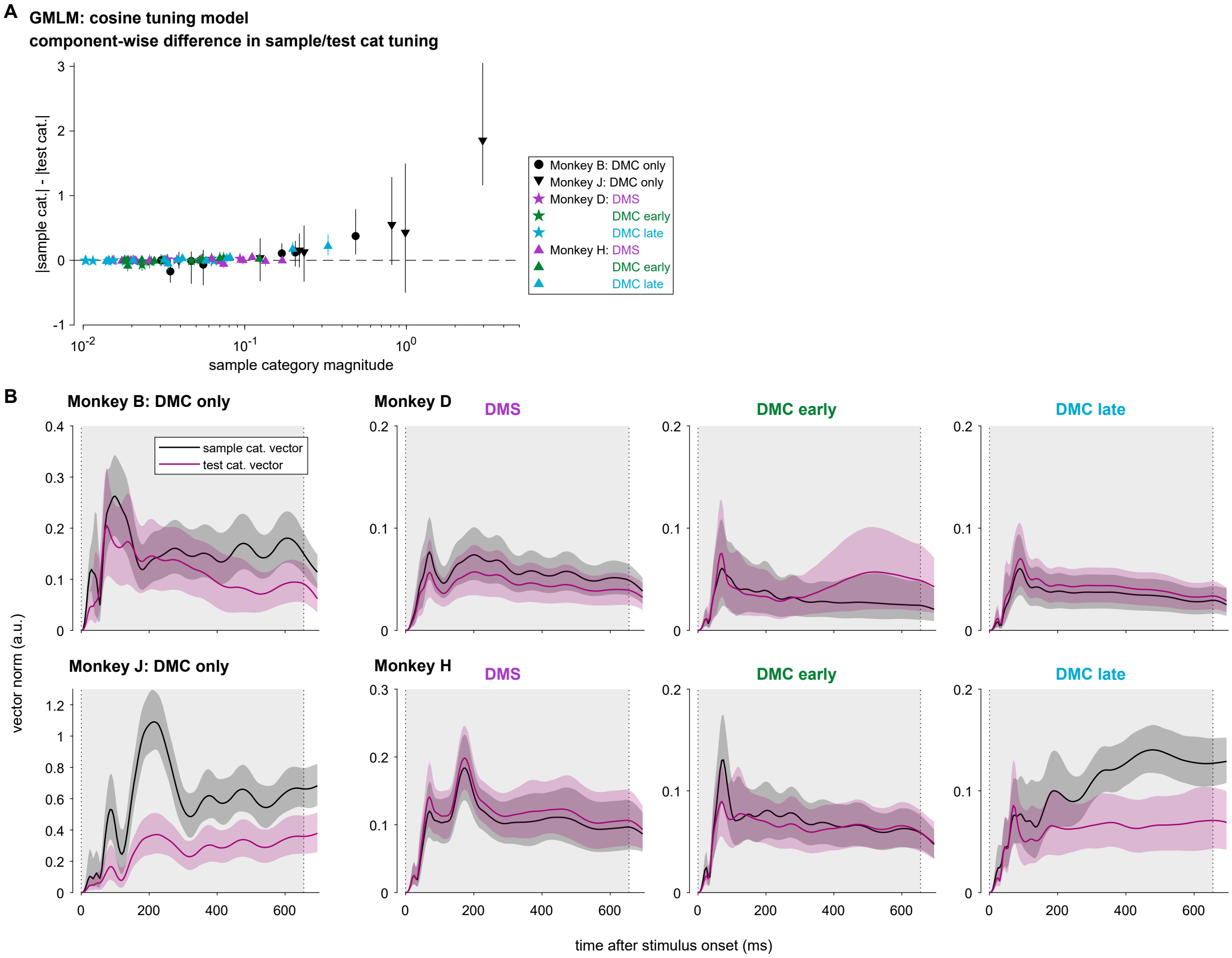

Supplementary Figure 14: Comparison of the sample and test category tuning of the low-dimensional GMLM cosine tuning model components. **a**, The difference in the magnitude of coefficients for the sample and test categories in the individual GMLM components (one point per each GMLM stimulus component per population). The component-wise sample category magnitude is computed as $C^{(r)}_{\mathrm{sample}}$ and the difference is $C^{(r)}_{\mathrm{sample}} - C^{(r)}_{\mathrm{test}}$ in Eq. 62 (see Methods). The points show the posterior median and the error bars denote a 99 % credible interval (50 000 posterior samples). **b**, The norm of the category tuning vector as a function of stimulus onset time for the sample and test stimuli in each of the eight LIP populations. The category vector norm is given in Eq. 60 (see Methods). The traces show the posterior median and the shaded regions denote a pointwise 99 % credible interval.

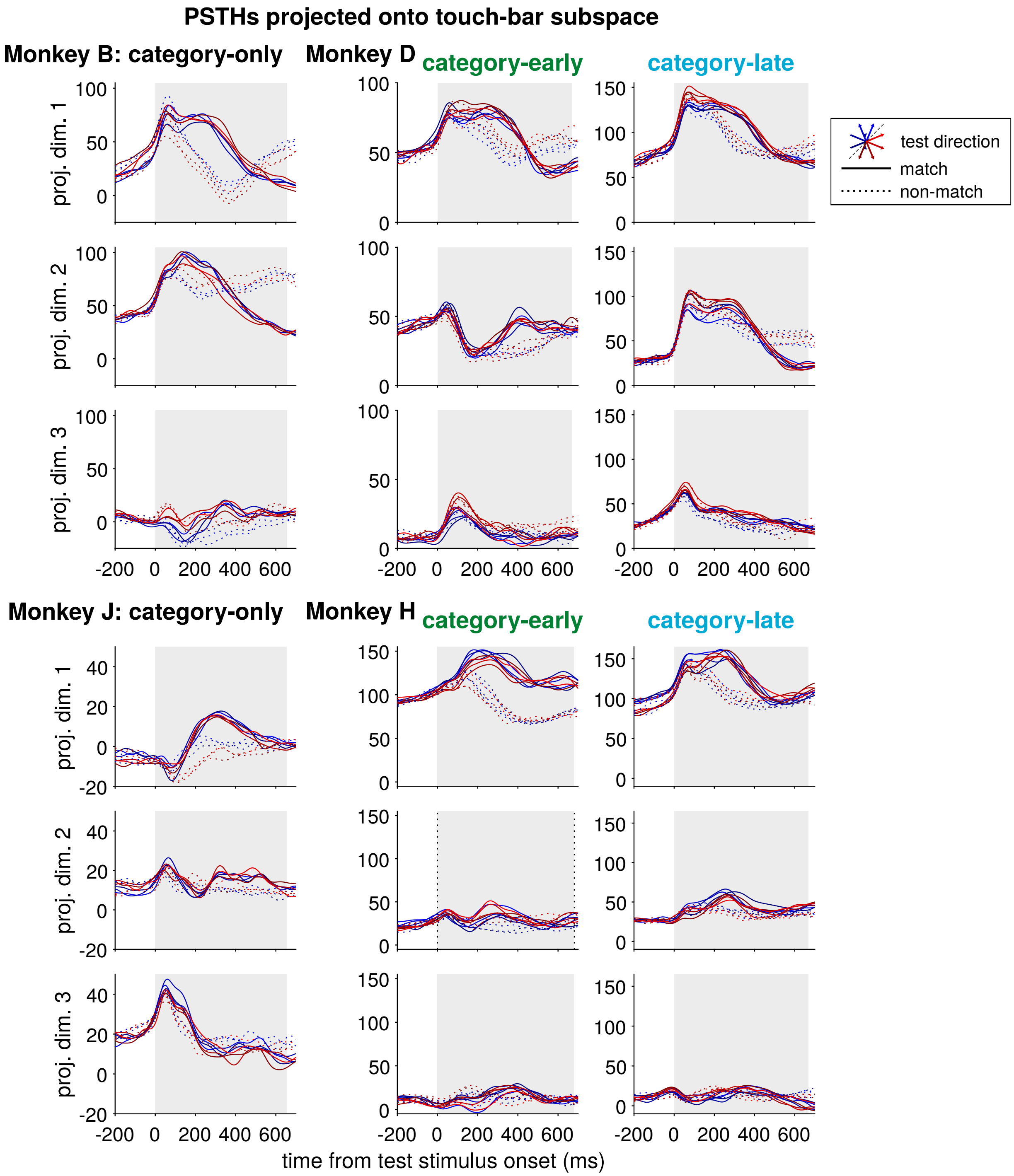

Supplementary Figure 15: The touch-bar response subspace does not encode stimulus category. The PSTHs of the six categorization populations projected onto the three-dimensional touch-bar subspace fit by the full GMLM (the subspace given by $\mathrm{orth}(\mathbf{V}^{\mathrm{tbar}})$, see Methods). The PSTHs are conditioned by both test stimulus direction (color) and by match (solid lines) or non-match (dotted lines) trials. The gray region denotes the stimulus presentation period (although it is terminated early on match trials by the touch-bar release).

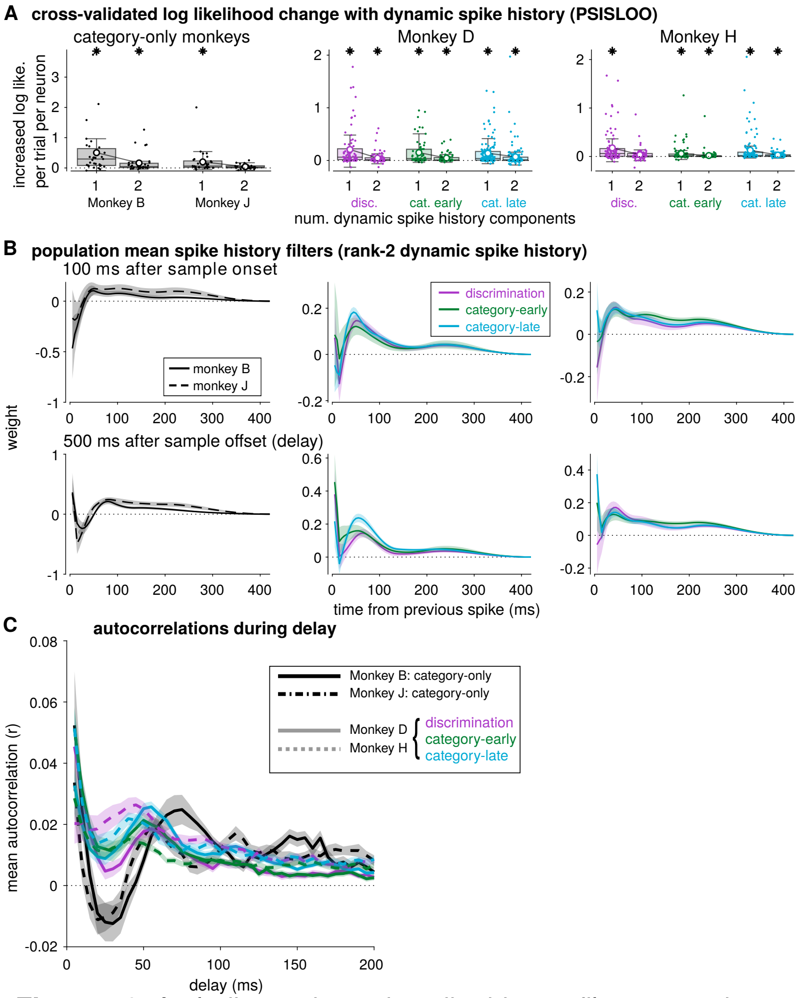

Supplementary Figure 16: Including a dynamic spike history filter tensor improves model fit. **a**, The mean change in cross-validated log likelihood per-trial for each neuron as a function of the number of components (i.e., the average improvement in predictive performance for adding an additional dynamic spike history component). The log likelihood for the rank-1 dynamic spike history is relative to the GMLM without any dynamic spike history (but still includes each individual neuron's static spike history filters). Leave-one-out cross-validation was estimated for each trial using Pareto-smoothed importance sampling (PSISLOO-cv). Stars denote a statistically significant improvement after including the dynamic spike history component ($p < 10^{-4}$, paired, one-sided Wilcoxin signed-rank test; number of neurons: B $n = 31$, J $n = 29$, D-disc. $n = 81$, D-early $n = 63$, D-late $n = 137$, H-disc. $n = 89$, H-early $n = 106$, D-late $n = 114$; Rank 1: B $p = 2.60 \times 10^{-6}$, J $p = 1.24 \times 10^{-5}$, D-disc. $p = 5.63 \times 10^{-13}$, D-early $p = 2.38 \times 10^{-10}$, D-late $p = 1.58 \times 10^{-18}$, H-disc. $p = 1.30 \times 10^{-10}$, H-early $p = 2.19 \times 10^{-11}$, D-late $p = 1.19 \times 10^{-14}$; Rank 2: B $p = 3.07 \times 10^{-5}$, J $p = 1.07 \times 10^{-3}$, D-disc. $p = 1.14 \times 10^{-7}$, D-early $p = 2.30 \times 10^{-7}$, D-late $p = 7.43 \times 10^{-11}$, H-disc. $p = 2.90 \times 10^{-4}$, H-early $p = 2.26 \times 10^{-6}$, D-late $p = 3.90 \times 10^{-13}$). The lines with white circle markers denote the average log likelihood per trial across neurons. The box plots show the median and 25 and 75 % quartiles over neurons and the whiskers extend to a 1.5 interquartile range from the edges. While the improvement after including two dynamic spike history components was often statistically significant, it was less dramatic than the gain from a single component. **b**, The mean population mean effective spike history filters for all eight LIP populations during sample stimulus presentation (top; 100 ms after stimulus onset) and during the delay period (bottom; 500 ms after stimulus offset). The spike history filters were computed as the MAP estimate of the GMLM with rank-2 dynamic spike history ($R_h = 2$). Error regions denote ± 2 SEM. **c**, The mean autocorrelation of the spike trains during the delay period (800 ms to 1600 ms after stimulus onset) for each population shows differences between the two pairs of monkeys. The colors and dashed or solid lines denote populations. The error region shows ± 1 SEM of the mean over neurons. The autocorrelation was computed separately for each cell over trials from each category and averaged over the two categories.
